# Editorial: Insights in head and neck cancer: 2021

**DOI:** 10.3389/fonc.2023.1180965

**Published:** 2023-03-28

**Authors:** Willem Lybaert, Jan B. Vermorken

**Affiliations:** ^1^ Multidisciplinary Head and Neck Cancer Clinic at the Antwerp University Hospital (UZA) and VITAZ, Sint-Niklaas, Belgium; ^2^ Faculty of Medicine and Health Sciences, Antwerp University (UA), Antwerp, Belgium; ^3^ Department of Medical Oncology, Antwerp University Hospital (UZA), Edegem, Belgium

**Keywords:** targeting p53, artificial intelligence diagnostic radiology, biomarkers immunotherapy, nasopharyngeal carcinoma, papillary thyroid cancer, parotid tumors

In recent years, a large oncological focus was set on the venue of immunotherapy in the systemic treatment of recurrent/metastatic squamous cell carcinoma of the head and neck (SCCHN), with a recent 4-year update of the pivotal KEYNOTE-048 study in the Journal of Clinical Oncology (1) first-line pembrolizumab and pembrolizumab + chemotherapy continued to demonstrate survival benefit *versus* cetuximab + chemotherapy in recurrent/metastatic SCCHN. Cetuximab as targeted therapy + chemotherapy stays an important treatment option in case of Combined Positive Score (CPS) < 1 or in second line after pembrolizumab.

However, immunotherapy in recurrent/metastatic disease benefits roughly 20% of patients in the long term. Immunotherapy has also the potential to improve the efficacy of treatment in the earlier disease settings. Although the outcome of several trials are to be awaited, the first reported trials in patients with locoregionally advanced SCCHN have not been too promising (2–4). Other approaches are therefore urgently needed in the field of SCCHN; the five following articles in Frontiers of Oncology all try to contribute to this scope, all in different domains of the disease.

Apart from the EGFR (5) - and PIK3/AKT/mTOR-pathway (6), much interest is settled on the p53 tumor suppression gene mutation, which is one of the most frequent genetic alterations in SCCHN. In the article of de Bakker et al., therapeutic strategies targeting p53 in SCCHN are divided into three categories related to three subtypes: reactivating wild-type (WT) p53, targeting mutated p53 by restoring WT conformation, and treatments that specifically affect HPV+ cancer cells by targeting the viral enzymes E6/E7, which are responsible for breakdown of p53. A lot of compounds are tested already *in vitro* and *in vivo* models, with some promising signals of activity, in combination with radiotherapy and/or chemotherapy. Some clinical studies are running at this moment, but like correctly stated by the authors, more research is needed to find more specific, safe, and effective p53 reactivators to improve the treatment of SCCHN. Let us hope that, in the near future, p53 becomes a second “undruggable target is now targetable” story like the KRAS G12C mutation with sotorasib (7) and adagrasib (8).

Artificial intelligence (AI) is the ability of a computer program or a machine to perceive, synthesize and infer information, as opposed to intelligence displayed by, for example, radiologists and pathologists. It is also a field of study that tries to make computers “smart”. They work on their own without being encoded with commands. The field of AI is more and more knocking on the door of SCCHN and is the subject of the other four fascinating studies (Qin et al.; Shi et al.; Zheng et al.; Jin et al.)

Clinico-radiological factors at baseline such as sex; age; T-, N-, and M-stage; overall stage; pathological subtype; and EBV copy number as well as the treatment plan are all used to estimate the long-term outcome in nasopharyngeal carcinoma (NPC). However, these factors are not perfect when it concerns the prediction of outcome and selection of optimal therapy for the individual patient. Qin et al. showed in a prospective study on 97 newly diagnosed NPC stage II–IV patients the value of different diffusion and perfusion parameters on magnetic resonance imaging (MRI) [the apparent diffusion coefficient (ADC), the pure diffusion coefficient (D), the pseudo-diffusion coefficient D*, and the perfusion fraction (f)]. Qin et al.’s conclusion that the perfusion parameters ADC and D together with the diffusion parameter D* could act as useful factors for predicting long-term outcomes and selecting high-risk patients with NPC seems reasonable, taking into account the mechanistics of diffusion and perfusion in the tumor environment. Unfortunately, the correlation between these parameters and histological characteristics was not studied in these patients. That knowledge in a larger cohort of patients is essential to understand the prognostic role of MRI-based parameters in NPC. Also, the consequences for (de-)intensifying treatment in function of this knowledge remain an open question for the clinician.

In the field of papillary thyroid carcinoma (PTC), an important issue is to assess the risk of central cervical lymph node metastasis (CCLNM) and related prognosis, risk of recurrence, and surgical management. Shi et al. showed in their retrospective study that their XGBoost model with 11 radiomics features used to calculate a radiomics score on ultrasound images of PTC in combination with capsular invasion, diameter, age, and calcification could predict CCLNM in patients with PTC, and that the model surpassed the evaluation ability of the radiologists. The model integrated thus ultrasound imaging information with clinical parameters of PTC patients. Models like XGBoost pave the way for more refined preoperative evaluation of CCLNM. However, prospective data are needed, and adding ultrasound images of the peritumoral environment and other radiological factors like location of the thyroid lesion are important for CCLNM risk estimation.

A simular setup of radiomic analysis on multi-phase computed tomography (CT) to differentiate parotid basal cell adenoma (BCA) from pleomorphic adenoma (PA) is shown in the retrospective study of Zheng et al. Fine needle aspiration biopsy is commonly done in the preoperative discrimination of parotid tumors, but its diagnostic yield is variable. This study provides a detailed analysis of the arterial and delayed CT-scan phases and different radiomic models that differentiate BCA from PA. Combining classical clinico-radiological predictors and radiomic features showed the best diagnostic performance in this study. Also, here, before clinical application, multicenter and prospective studies with larger datasets are needed.

To end with the beginning, an immense search for biomarkers for immunotherapy efficacy apart from PD-L1 scoring and tumor mutational burden (TMB) is ongoing. The study by Jin et al. is integrating an analysis of prognostic gene signatures and immune microenvironment in tongue squamous cell carcinoma. Many prognostic genes were accurately selected and established clusters with different survival rates using a series of screening methods. This article is one step in the direction of combined gene expression profiling and immune environment dissection of pathology specimens, but which genes and which innate and adaptive immune response cells are critical for the best immunotherapy effect in the individual patient, and is, at this moment, far from clear and not ready for daily clinical practice.

In summary, these five articles are inspiring us to more and more integrate AI tools in radiology and pathology of SCCHN and combining them with the skills of dedicated head and neck radiologists and pathologists. Both are going hand in hand together and open the way toward more personalized diagnosis, staging, prognosis, treatment planning, and more druggable targets like p53. We congratulate all authors for their valuable contribution to a better care for our patients. Having said this, more prospective studies in larger patient groups with uniform clinico-radiological standards and standardization of AI learning machinerie are essential before these techniques can be adopted in our clinic. Hard endpoints like local/regional relapse-free survival, progression-free survival, overall survival, and cost-effectiveness need to be included to validate these techniques for our patients.

## Author contributions

All authors listed have made a substantial, direct, and intellectual contribution to the work and approved it for publication.
